# “I don't want financial support but verbal support.” How do caregivers manage children's access to and retention in HIV care in urban Zimbabwe?

**DOI:** 10.7448/IAS.17.1.18839

**Published:** 2014-05-09

**Authors:** Joanna Busza, Ethel Dauya, Tsitsi Bandason, Hilda Mujuru, Rashida A Ferrand

**Affiliations:** 1 London School of Hygiene and Tropical Medicine, London, UK; 2 Biomedical Research and Training Institute, Harare, Zimbabwe; 3 Department of Paediatrics, University of Zimbabwe, Harare, Zimbabwe

**Keywords:** Zimbabwe, children, ART, access, adherence, social support, qualitative methods

## Abstract

**Introduction:**

Children living with HIV experience particular challenges in accessing HIV care. Children usually rely on adult caregivers for access to care, including timely diagnosis, initiation of treatment and sustained engagement with HIV services. The aim of this study was to inform the design of a community-based intervention to support caregivers of HIV-positive children to increase children's retention in care as part of a programme introducing decentralized HIV care in primary health facilities.

**Methods:**

Using an existing conceptual framework, we conducted formative research to identify key local contextual factors affecting children's linkages to HIV care in Harare, Zimbabwe. We conducted semi-structured interviews with 15 primary caregivers of HIV-positive children aged 6–15 years enrolled at a hospital clinic for at least six months, followed by interviews with nine key informants from five community-based organizations providing adherence support or related services.

**Results:**

We identified a range of facilitators and barriers that caregivers experience. Distance to the hospital, cost of transportation, fear of disclosing HIV status to the child or others, unstable family structure and institutional factors such as drug stock-outs, healthcare worker absenteeism and unsympathetic school environments proved the most salient limiting factors. Facilitators included openness within the family, availability of practical assistance and psychosocial support from community members.

**Conclusions:**

The proposed decentralization of HIV care will mitigate concerns about distance and transport costs but is likely to be insufficient to ensure children's sustained retention. Following this study, we developed a package of structured home visits by voluntary lay workers to proactively address other determinants such as disclosure within families, access to available services and support through caregivers’ social networks. A randomized controlled trial is underway to assess impact on children's retention in care over two years.

## Introduction

As antiretroviral treatment (ART) for HIV is scaled up across sub-Saharan Africa, a growing body of evidence highlights numerous challenges to timely uptake and optimal adherence among those eligible for treatment [1, 2]. Barriers to treatment initiation and longer-term retention in care have been documented across the “treatment cascade” comprising HIV testing, eligibility screening, treatment enrolment, adherence to drug regimens, lifelong participation in clinical monitoring and, where appropriate, social support services [3, 4]. Successful progression through the treatment cascade has been linked to a range of individual, community and structural factors, resulting in the use of socio-ecological perspectives in the analysis of barriers to retention in both care and development of interventions to facilitate each stage of treatment [5, 6].

For the most part, however, interventions have focused on adults’ engagement with HIV services. Two recent reviews note the lack of evidence on overcoming barriers confronted by children in initiating and sustaining HIV treatment, most of whom remain reliant on adult carers whose involvement is thus crucial in ensuring children's retention in care [7, 8]. Several studies suggest that family centred and community-based approaches that provide psychosocial support and links to other social benefits improve children's health-seeking and clinical outcomes [9–11]. The success of such interventions depends on their adaptation to the local context and ability to target the most relevant barriers to children's engagement in the treatment cascade. HIV-positive children's entry and retention in care will be shaped by factors specific to their family, community and wider social environment. The process of designing effective support mechanisms, therefore, includes identification of existing facilitators, as well as the barriers that will need to be targeted [12–14].

Skovdal *et al*. [15], have developed a conceptual framework that draws together findings from studies conducted in resource-limited settings, and categorizes commonly identified determinants of retention in HIV care into *material*, *symbolic*, *relational* and *institutional* dimensions (Table 1) [15].

**Table 1:** Dimensions of retention in care

Dimension	Social and contextual determinants of retention in care
Material	Poverty Availability of adequate food Distance to facilities Transport availability and cost Opportunity costs of attending appointments Treatment costs
Symbolic	Perceived stigma in the community Power balance between men and women Trust in biomedicine Competing medical discourses
Relational	Relationship with health staff Disclosure to child/family/community Support networks Social cohesion
Institutional	Health system capacity Drug stock-outs Quality of care (waiting times, confidentiality, privacy) Supportive institutions (CBO, churches)

Adapted from Skovdal *et al*. [15].

We used this framework to investigate contextual factors affecting HIV-positive children's engagement with HIV care in Harare, Zimbabwe. Despite declines in HIV prevalence in recent years, the 2010–11 Demographic Health Survey (DHS) found 15% prevalence among adults aged 15–49 [16] and 138,642 children under the age of 15 were estimated to be living with HIV in 2010 [17]. Currently, HIV testing of children remains patchy, initiated by nurses in primary care clinics if the child presents with signs of advanced HIV infection. Paediatric care is usually obtained through hospitals despite increasing decentralization of access to ART for stable adult patients. Although HIV treatment is freely available through public services, other medications incur costs and the system suffers equipment and staff shortages.

Our study was thus underpinned by concern that up to 80% of vertically infected children living with HIV in Zimbabwe remain undiagnosed [18, 19] and thus innovative strategies are required to improve their HIV testing rates and subsequent linkage to and retention in care. The primary aim of this study was to inform the design of a community-based intervention to support caregivers of children found HIV-positive, which is now being evaluated through a randomized controlled trial to assess its effect on children's retention in HIV care. We explored the barriers and facilitators experienced by caregivers of HIV-positive children in sustaining HIV treatment and care in a context of deep and sustained poverty.

## Methods

We used the Skovdal *et al*. [15] conceptual framework as an assessment tool, gathering information for each of its dimensions to build up a picture of locally salient material, symbolic, relational and institutional determinants of children's retention in care. We also sought to identify and map existing community-based initiatives that provide adherence support or similar outreach to families of HIV-positive children to avoid duplication and integrate our intervention within available support mechanisms to create synergy and strengthen access.

The study was conducted between October and November 2012 at the Harare Central Hospital HIV clinic, which has provided HIV diagnosis and care to children since the inception of the National Paediatric HIV treatment programme. Children who have been diagnosed with HIV are referred to this clinic for ART eligibility screening, initiation of treatment, and all follow-up care. Children continue to rely on the hospital for their clinical monitoring and ART drug supply until they transition to adult care. The hospital serves the same high-density suburbs in Harare that make up the catchment area of our planned intervention trial, making it likely that participants would reflect the socio-demographic profile, economic status, and living conditions of the trial communities.

We conducted semi-structured interviews with primary caregivers of HIV-positive children aged 6–15 years enrolled at the clinic for at least six months, followed by interviews with key informants from any community-based organizations (CBO) mentioned by caregivers as providing adherence support or related services in the hospital's catchment area. The 6–15 age group was selected because it includes children eligible for paediatric HIV care; younger children are the responsibility of maternal health programmes and those diagnosed after age 15 are classified as adults. Caregivers were sampled to reflect diversity by relationship to the child(ren) for whom they care, and background characteristics including age, sex, and residence. Interviews were conducted in a private room at the hospital while caregivers were waiting for an appointment. The topic guide addressed experiences of supporting children in health-seeking, diagnosis, disclosure (if applicable), attending regular appointments, undertaking medical tests, taking medication and positive living more broadly (mitigation of stigma, relationships at school, nutrition, psychosocial care, etc.) CBO key informants were interviewed at their workplace about the services they provided and their perceptions of how to successfully support adherence.

All interviews were conducted in Shona by four fieldworkers (three female and one male) with prior qualitative research experience who were further trained on the study tools and helped to pilot them. Fieldworkers transcribed their own interviews and translated them into English. The first author conducted thematic data analysis that organized excerpts from interview transcripts into the four structured dimensions of the Skovdal *et al*. conceptual framework, allowing for additional categories to emerge directly from the data to create new dimensions or refine their attributes.

Ethical approval for the study was granted by the London School of Hygiene and Tropical Medicine (UK), Biomedical Research & Training Institute (Zimbabwe), the Medical Research Council of Zimbabwe and the Harare Hospital Ethics Committee. Fieldworkers obtained written informed consent from all study participants prior to interview and emphasized that enrolment would not affect access to services at Harare Hospital.

## Results

We conducted interviews with 15 primary caregivers of HIV+ children and nine key informants from five locally active CBO that worked with families affected by HIV or vulnerable groups more generally (orphans, women in need of livelihood support). Among caregivers, we interviewed 12 women (eight biological mothers, two aunts, one grandmother and one cousin) and three men (all biological fathers). Seven of the nine CBO respondents were female, both paid staff and volunteers, for example, home-based care providers and organizers of community gardens to increase HIV-affected families’ access to vegetables.

Table 2 summarizes our findings for each dimension of our guiding conceptual framework. The determinants displayed in bold indicate those that emerged as particularly salient. We have added a further two determinants (family composition and stability; school-related experiences) and specified an additional component of quality of care (staff absenteeism) as these featured prominently in our data. Illustrative quotations have been selected to reflect diversity in experience and perceptions expressed, while the subsequent narrative section provides an overall synthesis of results. We did not identify many differences between information provided by caregivers and that from CBO informants, although caregivers focused on their personal experience while CBO respondents generalized their observations from the many families with whom they had worked.

**Table 2:** Locally prioritized issues affecting retention in care

Dimension	Social and contextual determinants of retention in care	Local characterization of determinants: excerpts from interviews
Material	Poverty Availability of adequate food Distance to facilities **Transport availability and cost** **Opportunity costs of attending appointments** Treatment costs	There is a grandmother who is taking care of more than 7 orphans that were left behind by her sons and daughters. Therefore, she experiences serious challenges in finding bus fare for the person who will take the children to the hospital when it's time to collect their medication. (Female CBO informant) You will find that a child is supposed to go for review but he does not have bus fare …. I would say the major challenge would be transport when they are going to collect their medication. (Female CBO informant) They were saying I should book [and] then I would be given a date to come and spend the whole day here. That did not go well with me. I cannot spend the whole day …. I am the bread winner, I have to provide for the family. This child is not the only one who should survive, I have other kids who also want to survive. (Male caregiver, father)
Symbolic	**Perceived stigma in the community** Power balance between men and women Trust in biomedicine Competing medical discourses	The disadvantages are that if you tell someone [the child's status] some women are not able to keep secrets. They might tell their young children who would then scold [the child] …. “You are HIV positive.” Therefore [the child] might feel offended. (Female caregiver, mother) They do it behind your back, spreading rumors. What makes a child get affected are the reactions of the community if her status is known by everyone. They will start to discriminate about her status and others will refuse to play with her. I am not saying it happened to my child, but they are just stories I hear people talking about. During these conversations you get to see people's reactions … the same reaction may happen to you, so I often keep quiet about my status. (Female caregiver, aunt) Long back when a person was positive people would laugh at them, saying this and that. But now it's OK. Now people have been educated and this is the status that most of the people are living with … most households have the same disease. And because people have been educated about it there is no one who can say anything bad about it. (Male caregiver, father) [My sister-in-law] no longer wanted to touch utensils that I was also using …. If my child was eating his food and shared [with another child], he would be harassed …. You could actually see that this person is discriminating against the child – probably she thought that that this is how one gets infected. But things changed when she had knowledge … a Mai Chisamba [popular TV host] talk show program about people living with HIV greatly assisted people in my family …. These people changed when that program was done. (Female caregiver, mother) People now have the knowledge about caring for HIV+ people so now their attitudes have changed also, but before that you would find in a household there will be plates, cups or spoons that would be labeled “do not touch.” (Female caregiver, mother)
Relational	Relationship with health staff **Disclosure to child/family/community** **Support networks** Social cohesion *Family composition and stability	This should be kept among us. I had to tell other people in the family to get support for taking her medication when I am not around. As a family they should know that [the child] is supposed to take her medication at 7. They should know that once it's 7 they should ask [her] to come and take her medication. This is why I had to disclose to the family. (Female caregiver, aunt) Some of the children haven't been properly disclosed to and they have got a lot of questions that have not been answered. And probably it goes back to the relationship with the caregiver. Do they even have time to sit down, talk and discuss what is happening? (Female CBO informant) [There are] those who have declined [to disclose to family] and they can tell us to talk to them alone. “Do not show it when you get to our house.” Some families are not free and we cannot just barge in asking to see so-and-so's mother. They can actually tell you that when you get to our house, “you can't see me after this” because they are not free. (Female CBO informant) The support I mostly need from my relatives? I don't want financial support but verbal support – to comfort with words, it's a very vital form of support such that it makes me as the caregiver continue persevering and not give up because of the support I will be getting from my family …. If a person get supports such that they wake up to people asking them “how are you doing? Have you eaten? Have you taken your medication?” a person can recover just from that. (Female caregiver, aunt) When I leave her, there is one of her aunts who is married and stays close to home, she is the one I leave her with … I explained to [the aunt] that she takes her medication at such a time while she is there. the aunt actually sets her alarm that rings every day. (Female caregiver, mother) The uncles help, … they help in buying things … if they have money, but if they do not have money we face a challenge in getting things she might need or want …. If I have travelled or when I'm not around, they make sure they monitor her saying, “did you drink your medicine?” since she is a child she can get carried away and forget. (Female caregiver, grandmother) I think it varies from case to case …. Some children are staying with their biological parents. Some are staying with grandmothers. Some are staying with extended family members who are taking care of them. So the treatment they all get differs because you will find that their categories are different. Some are well catered for …. And some caregivers do not accept the children. They are just staying with the child because they do not have options since the child was brought there. (Female CBO informant) Someone might have lost her mother, and her father marries another woman, her stepmother. The stepmother stigmatizes the child. She does not take care of the child. We have a case of a child who was left in the care of her aunt, her father's brother's wife who does not like her at all. (Female CBO informant) He [child's father] is also in the program. He is positive. So it was difficult for him to care for the child and care for himself and he now has another wife. The wife refused to accept the situation so my mother [child's aunt] took the child and said we are now staying with her. (Female caregiver, cousin) Those children staying with people who are not their biological parents but are guardians, those are the ones with problems because they may want to hide or not really explain what the child might be suffering from … At times [children] will be told they are no longer wanted at that house or at times they are constantly moving from one house to the other and changing guardians. (Female CBO informant)
Institutional	Health system capacity **Drug stock-outs (Cotrimoxazole)** **Quality of care** (waiting times, confidentiality, privacy, *staff absenteeism) Supportive institutions (CBO, churches) *School-related experiences	Last week there was a child who came saying that she had been to [a Hospital] but there were no drugs. She could not get Cotrimoxazole because it was out of stock and she didn't have money. Therefore this is another challenge that we face. (Female CBO informant) He won't take Cotri for a full month, really, but you know it's just one of those moments when you have to create a plan for survival, like I say he will drink Cotri today, but if he drinks Cotri today he skips the next day and drinks the day following. (Female caregiver, mother) If you come early to the pharmacy it doesn't really matter, you will have to wait because at times the pharmacist will be on his break or the drugs have not yet been delivered at the hospital. You might be given the drugs at 11 am … honestly there are just too many people here, such that every day from Monday to Thursday the numbers will be many. That is why they close on Friday – to take a break from the numbers … because it's just too much for them … (Female caregiver, mother) Sometimes the service is poor. I come in the morning, like I came around 8 but I might still be here until 1 pm. The pharmacist leaves for tea break and he does not come back. You will wait. Sometimes we are told there are no drugs in the pharmacy and he has to collect them from the main pharmacy. You can wait for him for more than an hour … It is difficult. You would have come early but the time that you leave … These are some of the things that are happening here. Their service is not good, especially the pharmacist … It was already after 1 pm. He had gone for tea and did not come back and people were queuing from that end to this end. (Female caregiver, grandmother) Stigma in school is really difficult to measure, it has actually lessened because teachers are more knowledgeable about HIV, but you find that kids do not take their medication at school because kids being kids say a lot of hurtful and negative stuff about each other, so you find our kids telling us they want to drink their pills at 6 am because at school other kids will laugh at them. (Male CBO informant) Some teachers discriminate when [the children] go outside to drink their medication. The teachers would then say, “they have gone to drink their medication for AIDS.” (Female CBO informant) Or maybe someone is at a boarding school where one will be drinking pills every day. Then the other will start being inquisitive – why is this one taking pills every day? They will say “this one has AIDS.” (Female CBO informant) I went back the following day and the teacher was briefed by the headmistress. I explained that this is the child's condition. I am doing this so that you are aware of her status. She might be slow in class or do certain things just because she is sick. You are then supposed to follow her pace. And the teacher said it's OK. I am grateful she is still with the same teacher. (Female caregiver, aunt) The reason is that some people …. they will just publicize, publicize. The child will end up not relating well to his peers at school. We felt it's better to leave things as it is [by not telling any school staff about the child's status]. (Male caregiver, father) Yes the headmaster accepted it … he then encouraged me to continue looking after my grandchild properly … I have no problems really, when it is time to go collect her medication she informs her school mistress that she is not coming to school, she is going to collect her medication or to see the doctor. (Female caregiver, grandmother)

Adapted from Skovdal *et al*. [15].

* Items marked by bold italics have been added based on study findings.

Among material considerations, respondents saw distance to facilities and cost of transport as synonymous; caregivers who lived within walking distance of the hospital felt grateful, while 9 of the 15 caregivers mentioned difficulty affording regular travel to bring children to appointments or to collect medicines. Opportunity costs included taking time away from income generating activities and risking loss of employment for those with formal jobs. Although CBO staff raised pervasive poverty as a cross-cutting issue, caregivers did not discuss it in general terms, focusing on specific financial difficulties, for example, paying school fees. Food insecurity was mentioned among these but did not appear to affect children's adherence to medication.

Among symbolic determinants, perceived stigma emerged as a significant concern and is probably the greatest barrier to progression through the treatment cascade in our study setting. Both anticipated and experienced discrimination clearly affected children's retention in care. Caregivers refrained from disclosing their own or a child's status out of fear of repercussions, giving numerous examples of discrimination they had witnessed. Two respondents observed that HIV-related stigma had reduced in recent years, but most felt reluctant to inform people, including other household members, even when they had not directly experienced discrimination. Secrecy around a child's illness led to disruption of drug regimens, particularly when there were visitors in the house, and could also discourage participation in support groups or willingness to receive home-based care visits. On the other hand, respondents recalled cases where HIV-positive individuals had “given testimony” to raise local awareness as well as TV programmes portraying HIV as a manageable chronic condition such as diabetes.

There were clear links between perceived stigma and relational determinants. Anticipated discrimination affected whether or not disclosure occurred within and beyond families, influencing availability of support through social networks. When more family and community members knew the child's status, a greater pool of resources became available for caregivers to draw on for financial and logistical assistance, including borrowing money for transport, asking a relative to collect the child's drugs, or leaving the child in the care of a trusted adult who would ensure they took their medication as required.

We further found disclosure and reliance on others for support to be closely related to the family's composition and stability, which has been added as a relational determinant that will need to be addressed to facilitate engagement in care. Families experiencing dissolution or marital discord resulting in the child's mobility or change of caregiver experienced greater challenges in maintaining care. CBO staff reported that the nature of a child's relationship to others in the household played an important role. Interestingly, despite some demonization of “stepmothers” and concerns about non-parental guardians, CBO respondents agreed that distant relatives or extended family members could be as or more diligent in care of HIV-positive children than biological parents.

At institutional level, caregivers focused on their experiences of hospital and schools. Despite efforts to probe into other forms of institutional support such as churches, local NGO and CBO, or community saving schemes, few were mentioned. HIV services generally received praise, with particular appreciation for nurses. Caregivers liked that a meal was provided to children waiting for appointments, although long waiting times, the need to return regularly, and problems with pharmacy management caused difficulties. Shortage of cotrimoxazole (provided without charge in the National HIV programme) meant that children were given prescriptions requiring purchase from pharmacies. Many families were unable to afford the drug and either borrowed money or reduced the frequency of dosing. Furthermore, the on-site pharmacy appeared to have staffing shortages leading to exceptionally long waiting times.

A key finding is the critical role played by the school environment in shaping a child's experience of care. How a child was treated at school affected adherence to medication, ability to attend appointments and overall psychological well-being. Examples of supportive schools and helpful teachers contrasted sharply with stories of children harassed by fellow pupils, singled out for taking medication and identified as HIV-positive by teachers. Boarding schools were separately mentioned for posing challenges to privacy when taking medication.

## Discussion: from formative research to intervention design

At the time of this study, decentralization of paediatric HIV care was being introduced in Harare. Based on existing literature highlighting the dependence of children on caregivers for successful HIV care [10, 18–20], we planned to test an intervention providing family centred support alongside provision of new ART services in primary health clinics. Following analysis of this study, we developed a volunteer lay worker (VLW) programme based around 12 structured home visits using a case management approach (available as Supplementary file). VLW lead caregivers through a process of planning and implementing feasible care strategies for children's successful adherence and long-term engagement in HIV care. Through a set of standardized activities, VLW work with caregivers, HIV-positive children and the wider household to identify available skills, resources, support structures and coping mechanisms that might help overcome identified barriers and strengthen existing facilitators. Each home visit addresses one or more of the key social and contextual determinants identified through the formative research.

Children testing HIV-positive will be recruited into the trial, and randomly allocated to receive the VLW-delivered intervention, or to the control group. The decentralization of HIV treatment, however, will become the standard of care in all study clinics, and should itself mitigate well-documented concerns about treatment accessibility [21]. Table 3 details how we have used study findings to design the intervention. The main outcome is retention in care and adherence to ART (measured through HIV viral load suppression rates) after one year's follow-up.

**Table 3:** Translation of formative research findings into intervention components

Key determinant	Approach through VLW home visit programme
Transport availability and cost	• Provision of decentralized HIV care, allowing testing, eligibility assessment, treatment and follow-up to be accessed at one local site. A dedicated nurse in each clinic will help families navigate services. This is a novel approach for HIV-positive children and eliminates the need to travel to Harare Hospital, reducing transport time and costs.
Opportunity costs of attending appointments	• On-site testing offered by providers for all children aged 6–15, regardless of reason for coming to the clinic. No separate appointments will need to be made for testing, saving time and limiting loss to follow-up. • Testing, eligibility screening and treatment (when required) will be provided at the same clinic.
Perceived stigma in the community	• Provider-initiated testing for all children in the eligible age range attending the primary health clinics ensures families are not identified as seeking care from designated HIV clinics. • Widespread testing helps reaffirm the message being promoted throughout Zimbabwe that HIV is a chronic and manageable condition. • VLW home visits will use social network mapping to help caregivers identify community members whom they trust, to encourage greater openness when appropriate. • Education session for caregivers and any interested household members on HIV transmission, to dispel myths about infection through casual contact.
Disclosure to child/family/community	• Structured home visits facilitate discussions on disclosure to the child, with suggestions (and provision of materials) for how to disclose to children at different ages. • VLW offer to be present and assist with disclosure to children or other household members. • VLW provide encouragement for disclosure within the family over several home visits, linking it to being able to receive social support from others.
Support networks	• Social mapping activities help caregivers identify individuals on whom they can rely for different kinds of support, including psychosocial, practical and financial. VLW encourage proactive engagement with individuals identified as most supportive in the network. • Formal assessment of household needs lead to referrals to existing support groups and other organizations that support families affected by HIV. • VLW will provide direct support through giving advice on ART and clarifying information from health care providers, and the use of SMS reminders prior to scheduled appointments.
Family composition and stability	• Clarification provided to health care providers on national guidelines regarding guardianship of children, increasing the pool of caregivers authorized to consent to testing and initiate the child on treatment. • VLW home visits will assess the structure of the household and engage with members other than the primary caregiver where appropriate.
Drug stock-outs (Cotrimoxazole)	• All supplies are being provided through the programme, including HIV testing kits, cotrimoxazole and ART. • Supply chain easier to manage through six primary health clinics with smaller client load than through centralized hospitals.
Quality of care (waiting times, confidentiality, privacy, staff absenteeism)	• A dedicated nurse will be responsible for liaising with enrolled families at each of the six participating facilities, ensuring they progress through each stage of the testing and treatment process. • Additional training for clinic staff in provider-initiated testing and case management (linking clinical care with household support visits) will be provided. • Primary health care clinics are less over-burdened than the urban hospital and should be able to manage patient flow more efficiently.
School-related experiences	• Issues related to schooling will be discussed during household visits and disclosure to teachers or school administration raised as an option.

We acknowledge that not all barriers identified will be adequately addressed through home-based support, particularly material concerns. Given the widespread and intense poverty experienced in this peri-urban area, it is not surprising that many respondents highlighted their economic struggles and very real constraints in meeting family members’ needs. At the same time, however, it was clear that psychosocial and emotional support was perceived as a resource to help cope with and mitigate these difficulties. Our intervention aims to provide an initial source of direct emotional support and strengthen caregivers’ capacity to benefit from their social networks. However, we also will link families to material assistance where possible. We conducted a mapping exercise of relevant services offered in our study communities, for example, support groups, food supplementation and child protection. VLW will assess household needs and assist in linking caregivers to other organizations through referrals that will then be monitored. While the child's school environment can be explicitly discussed, the intervention will not directly provide outreach to educational institutions. Where caregivers feel there is a supportive environment at the child's school, the VLW will encourage and provide tips for disclosure as part of the structured session on increasing community support networks; in instances where schools are considered not to have an enabling environment for children in HIV care, however, the programme will have limited usefulness.

We explicitly limited the remit of the VLW intervention to maximize its future sustainability. Although the trial receives funding from the Wellcome Trust, the intervention was designed with the intention that it could be implemented within existing national guidelines and health service structures, with minimal additional costs. Home-based care services already exist in these communities and we have partnered with an experienced organization already working in the same area of Harare. Decentralization of HIV care is an on-going process and we hope the home visits and support from VLWs will increase its success and cost-effectiveness.

Our study had its own limitations. Although we selected the site to maximize similarities between respondents and proposed trial communities, there were some differences that could affect the dimensions explored. First, caregivers in our study were attending HIV services at Harare Hospital and thus successfully had overcome barriers in ensuring children's engagement in care. We did not capture the experiences of households with undiagnosed children or those lost to follow-up following treatment initiation. Similarly, CBO representatives based their narratives on households with which they were actively involved and were less able to characterize those that chose not to take up HIV-related support.

## Conclusions

Our study demonstrates how an existing conceptual model of retention in care and adherence to HIV treatment can be used as a rapid assessment tool to guide formative research for the development of a context-specific intervention. The Skovdal *et al*. [15] framework provided a useful means of classifying potential barriers and facilitators. By populating this framework through interviews with primary caregivers, HIV-positive children and key informants from CBOs engaged in community support, we developed a locally relevant community-based intervention to support families with newly diagnosed children remain engaged with HIV care.

Given pervasive concerns about distance to health services, the cost of transport and potential loss of income while seeking care, decentralization of HIV testing and treatment should itself increase identification and treatment initiation of previously undiagnosed HIV-positive children. Similarly, provider-initiated testing for children coming to the clinic for a wide range of concerns may help bolster national efforts to position HIV as a chronic condition similar to diabetes, rather than a life-threatening disease.

Based on targeting the identified barriers and strengthening enabling factors, the intervention provides structured activities used by VLW during a series of home visits. We produced a comprehensive VLW manual that details each activity and provides the requisite worksheets, checklists and notes for issues requiring follow-up to ensure VLW covers all scheduled content. This approach is likely to have several advantages. First, it standardizes the counselling messages, household assessments, and referrals provided to caregivers ensuring that VLW feel adequately prepared and guided through each visit. This also reduces the likelihood of VLW providing advice or addressing topics for which they are unqualified. Second, it increases the intervention's sustainability and potential expansion to new local settings if the trial proves successful, as the manual can be distributed widely, accompanied by a short, focused training course that does not need to rely on the trial's resources.

The trial of the intervention to support families is currently underway. We will conduct a process evaluation alongside the trial, including additional qualitative research over the course of implementation. This will assess whether families of newly identified HIV-positive children experience the predicted determinants of their engagement with care, and how well the VLW home-based visits are able to equip caregivers and others in their households to overcome the barriers they confront and strengthen facilitating factors.
